# Staminal hairs increase pollinator attraction and pollination accuracy in *Tradescantia fluminensis* (Commelinaceae)

**DOI:** 10.1093/aobpla/plad067

**Published:** 2023-10-01

**Authors:** Deng-Fei Li, Yi-Dan Chen, Zhen Liu, Ai-Ting Liang, Ju Tang, Xian-Chun Yan

**Affiliations:** Key Laboratory of Southwest China Wildlife Resources Conservation (Ministry of Education), China West Normal University, Nanchong 637002, China; Key Laboratory of Southwest China Wildlife Resources Conservation (Ministry of Education), China West Normal University, Nanchong 637002, China; Key Laboratory of Southwest China Wildlife Resources Conservation (Ministry of Education), China West Normal University, Nanchong 637002, China; Key Laboratory of Southwest China Wildlife Resources Conservation (Ministry of Education), China West Normal University, Nanchong 637002, China; College of Life Sciences, Anhui Normal University, Wuhu 241000, Anhui, China; Key Laboratory of Southwest China Wildlife Resources Conservation (Ministry of Education), China West Normal University, Nanchong 637002, China

**Keywords:** experimental manipulation, honeybee, pollen deposition, pollination accuracy, *Tradescantia*, visitation rates

## Abstract

Staminal hairs are the particular appendages of stamens, which may affect pollinator foraging behaviour and pollen transfer. However, experimental evidence of the functions of staminal hairs in pollination remains scarce. Here, we conducted staminal hair manipulation experiments in *Tradescantia fluminensis* (Commelinaceae) to investigate their effects on visitation and pollen transfer by bees. Our observations revealed that both visitation rates and visit duration of honeybees (*Apis cerana*) to control flowers were significantly higher than that of hairless flowers. Moreover, removing the staminal hairs significantly decreased pollen deposition by honeybees (*A. cerana*), but did not affect pollen removal. The staminal hair was similar in length to the stamen and the pistil of *T. fluminensis*. The staminal hairs provide more footholds for honeybees, and they lay prone on the staminal hairs to collect pollen, which increased the accuracy of pollination through the consistent pollen placement and pick-up on the ventral surface of honeybees. These results showed that the staminal hairs in *T. fluminensis* may represent an adaptation to attract pollinators and enhance pollination accuracy.

## Teaser

Staminal hair is a particular floral trait. Staminal hairs are generally moniliform and common in the Commelinaceae. In *Tradescantia fluminensis* (Commelinaceae), the white and conspicuous staminal hairs can attract honeybees and provide footholds for honeybees. Honeybees lie prone on the staminal hairs to collect pollen, and the pollen grains from the anthers are placed on its ventral surface in which contacts the stigma during the bee’s next flower visit, and this increases the accuracy of pollination. Consequently, staminal hairs in *T. fluminensis* represent an adaptation to attract pollinators and enhance pollination accuracy.

## Introduction

Almost 90 % of flowering plants are pollinated by animals (Ollerton *et al.* 2011), and some of them have evolved distinctive floral traits to attract pollinators (Duffy and Johnson 2015; Suetsugu *et al.* 2022). Floral traits affect pollinator foraging behaviour and pollen transfer and, thereby, influence the reproductive success of plants (Zhang 2011; Essenberg 2021; Li *et al.* 2022).

Floral hair is a common floral trait, which may occur on all floral parts. Pollination-related hairs occur mainly on the androecium. Staminal hairs are the particular appendages of stamens, and they are generally moniliform and common in the Commelinaceae (Faden 1992). In species of *Tradescantia*, staminal hairs often closely surround the yellow anthers, seemingly drawing attention to them. Knuth (1906) recorded the staminal hairs in *Tradescantia* as providing support and footholds for visitors, and such flowers were especially favoured by bees. The staminal hairs may also attract pollinators by being colourful and contrasting with the anthers (Faden 1992). However, these hypotheses (i.e. providing support and footholds for visitors and attracting pollinators) of staminal hairs have not been experimentally tested in species of *Tradescantia*.

In addition, the dense hairs of stamens in species of *Tradescantia* and *Cyanotis* may function further by interfering with pollen collection (Faden 1992). In order to increase male reproductive success, many species have several stamens and plenty of pollen. Due to the activities of pollinators, pollen loss during the process of pollen transfer is always very high, and normally less than 1 % of the pollen removed from the anthers can reach conspecific stigmas (Harder and Wilson 1994; Thomson *et al.* 2000). Owing to the remarkable pollen waste, the flowers of some species have evolved distinctive floral traits to control the amount of pollen output. In this case, a pollinator only removes a small amount of pollen in a single visit, and then more pollinators participate in the process of pollen transfer, thus reducing the pollen loss (Lloyd and Yates 1982; Harder and Thomson 1989). The staminal hairs may control the amount of pollen removed by the pollinator in a single visit. Sinclair (1968) mentioned that honeybees visiting *Tradescantia* flowers often draw together all of the anthers before collecting pollen, which might increase the efficiency of pollen collection. The density of the staminal hairs and their proximity to the anthers in *Tradescantia* and *Cyanotis* flowers may interfere both with the anthers being pulled together and with pollen being scraped or combed off them (Faden 1992). However, this function of staminal hairs is much less clear and can only be tentatively guessed. Further experimental evidence is required to test the hypothesis that staminal hairs may interfere with pollen collection by bees.

Plants attract pollinators to transport pollen to stigmas. Successful pollen transfer depends on stigma contact at the site on a pollinator’s body where pollen grains are placed (Armbruster *et al.* 2014). The structure of flower and the posture and behaviour of bees affect the placement of pollen (Tong and Huang 2018). The optimum geometry of flowers is one that causes anthers and stigmas to contact pollinators in the same location, promoting pollen transport to stigmas and pollen receipt by stigmas, respectively (Armbruster *et al.* 2009, 2014). Staminal hairs provide more surface area and more footholds for visitors. Staminal hairs may affect how and where pollinators move within the flower, which may influence pollen transfer. Therefore, we hypothesized that staminal hairs may help to make pollen placement and pick-up on pollinators be precise and consistent (i.e. pollination accuracy).

Here, we conducted staminal hair manipulation experiments to test the hypotheses related to how staminal hairs affect pollinator foraging behaviour and pollen transfer in *Tradescantia fluminensis*. This species is a cultivated plant originally from tropical America and widely cultivated as an ornamental in China. The staminal hairs of this species are white and conspicuous, and closely surround the yellow anthers. Based on field investigations, we aimed to address the following questions: (i) do flowers with staminal hairs increase visitation rates and visit duration of pollinators? (ii) do the dense hairs of stamens interfere with pollen collection by bees to control the amount of pollen removed? (iii) do staminal hairs enhance pollination accuracy or do staminal hairs increase the amount of pollen grains deposited on the stigmas?

## Materials and Methods

### Study species and site

**Figure 1.**
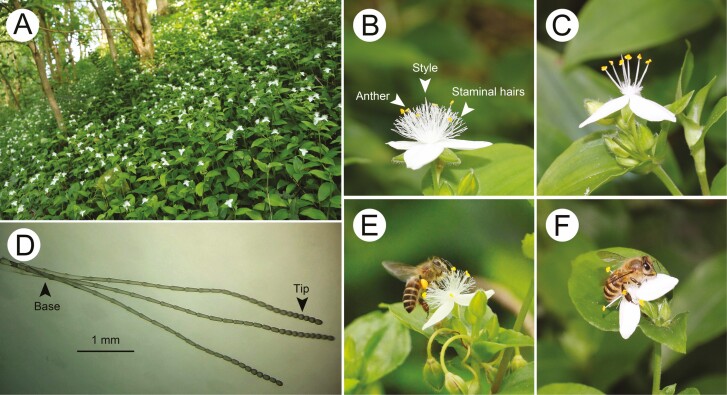
Staminal hairs in *Tradescantia fluminensis* and manipulated flowers. (A) A field population of *T. fluminensis*; (B) side view of an intact flower with white hairs (control); (C) a manipulated flower with all staminal hairs removed (hairless); (D) three staminal hairs of *T. fluminensis* under a light microscope, the base and the tip of each moniliform hair with long cells and round cells, respectively; (E) a honeybee *Apis cerana* lying prone on the staminal hairs to collect pollen on the intact flower; (F) a honeybee *A. cerana* lying prone on the petals to collect pollen on the hairless flower.

The genus *Tradescantia* consists of about 70 species predominantly occurring in tropical America, and some species were introduced in China. *Tradescantia fluminensis* (Commelinaceae) is a hermaphroditic perennial herb distributed in various shaded and moist habitats (Fig. 1A). Plants produce umbels. Each flower is actinomorphic, nectarless, with three white petals, one pistil and six stamens surrounded by dense staminal hairs (Fig. 1B and C). The flowers have a weak scent. The anthers are yellow and contrast with the white staminal hairs. The staminal hairs are moniliform (Fig. 1D). The base and the tip of each hair consist of long cells and round cells, respectively. Flowering generally occurs from April to June. Individual flowers remain open for one day.

Field investigations were conducted during the flowering season of 2023 on a slope under a forest of China West Normal University (30°83ʹN, 106°07ʹE; 221 m above sea level) in northeast Sichuan, Southwest China. The study area was approximately 10 m × 30 m.

### Measurements of floral traits

To understand the floral traits of *T. fluminensis*, 20 bagged flowers from different plants were examined. First, pistil length, stamen length, staminal hair length, petal length and width, sepal length and width, corolla diameter and pedicel length of each flower were measured with a digital calipre to 0.01 mm. Second, staminal hair number per flower was counted under a stereoscopic microscope JSZ6S (Nanjing Jiangnan Yongxin Optical Co. Ltd., Nanjing, China). To examine the production of pollen grains per flower, the anthers of each flower were collected in a 1.5 mL microcentrifuge tube. The anthers in each microcentrifuge tube were ground-up through using a steel-refined indentation pen with a round head 2 mm in diameter. Then 1 mL of 75 % alcohol was added to the microcentrifuge tube. The suspensions were stirred in a vortex mixer for 2 min, and five 1-μL samples of suspension were drawn on microscope slides, after which the number of pollen grains in the samples was counted under a light microscope at×40 magnification. Pollen count of the five sub-samples (1 μL each) was averaged and multiplied by the dilution factor (1000) to obtain the total number of pollen grains per flower.

### Effects of staminal hair removal on visitation by honeybees

Our field surveys showed that the Asian honeybee *Apis cerana* was the only visitor species to *T. fluminensis* during our experiments. To examine the effects of staminal hair removal on the visitation rates and visit duration of honeybees (*A. cerana*), at least 200 inflorescences of *T. fluminensis* were selected randomly from different individuals and were bagged with fine-mesh polyester bags before blooming in April and May 2023. Most of the flowers bloomed at noon, which may be due to temperature, light or other factors. When the flowers were in bloom, we selected 40 flowers from 40 inflorescences (each with one fresh flowers) to conduct manipulation experiments before 13:00 p.m. on each sunny day between 27 April and 11 May 2023. The experiments were conducted as follows: (i) all staminal hairs of 20 flowers were removed using a fine forceps (hairless, Fig. 1C); (ii) 20 flowers with no manipulation as a control (intact, Fig. 1B). For the control, we made a small cut on the petal to control for any effect of volatiles from tissue damage on honeybee behaviour. A total of 200 flowers (40 flowers × 5 days) were used in two treatments, with a total of 11 h of observations conducted for each treatment. All flowers were exposed to honeybees after the treatments. Subsequently, the visitation rates of honeybees (visits per flower per 15 min) were recorded in the period of 13:30–17:00 p.m. and their visit duration was recorded randomly.

### Effects of staminal hair removal on pollen deposition and pollen removal by honeybees

To examine the effects of staminal hair removal on pollen deposition and pollen removal, 60 flowers of *T. fluminensis* were selected randomly from different individuals and were bagged with fine-mesh polyester bags before blooming in May 2023. When the flowers bloomed, all staminal hairs of 30 flowers were removed (hairless), while the remaining 30 flowers with no manipulation were used as a control (intact). For the control, we also manually made a small cut to control for the effect of flower damage on pollen removal and deposition. All flowers were exposed to honeybees (*A. cerana*) after the treatments. When the flowers had been visited a single time in the field, we harvested the flowers immediately. To estimate pollen grains deposited on the stigmas of the flowers, each stigma was placed on a microscope slide and then squashed under a coverslip. The pollen grains were counted under a microscope. Moreover, we counted pollen grains remaining in the anthers of the flowers following the methods described above. Pollen removal per flower was calculated as the mean number of pollen grains per flower (see ‘Results’ section) minus the remaining grains per flower.

### Statistical analyses

The pairwise *t*-test was used to determine differences between the pistil, the stamen and the staminal hair length. In the staminal hair manipulation experiments, a generalized linear model (GLM) with normal distribution and identity-link function was conducted to identify differences in the visitation rates (with visitation rate as a dependent variable, and different treatments as factors) and the visit duration (with visit duration as dependent variable, and different treatments as factors) of honeybees to flowers between hairless and control. To compare pollen removal, pollen deposition and remaining pollen grains between the two treatments, data were analysed with a GLM with Poisson distribution and loglinear-link function, with pollen number as a dependent variable and different treatments as factors. All statistical analyses were performed in SPSS V. 19.0 (SPSS Inc., USA).

## Results

### Floral traits

On average, each *T. fluminensis* flower produced 81070 ± 4430 pollen grains and 219 ± 10 staminal hairs (Table 1). The flower is nectarless so that provides bees with a lot of pollen. The staminal hairs are moniliform, the tip of each hair consists of round cells that help the bees to cling (Fig. 1D and E). The pistil tends to be longer than the stamen (about 0.3 mm longer), and this difference is marginally significant (*t* = −2.07, *P* = 0.052; Table 1). The anther and stigma were likely to contact pollinators in the same location. The staminal hair length (5.97 ± 0.08 mm) was 0.72 mm and 0.92 mm shorter than the stamen and the pistil length (*t* = 5.017, *P* < 0.001 vs. *t* = 9.943, *P* < 0.001, respectively).

**Table 1.** Floral traits of *Tradescantia fluminensis*. *N* = number of sampled flowers.

Floral traits	Mean ± SE	*N*
Pistil length (mm)	6.99 ± 0.13	20
Stamen length (mm)	6.69 ± 0.13	20
Staminal hair length (mm)	5.97 ± 0.08	20
Staminal hair number per flower	219 ± 10	20
Pollen number per flower	81070 ± 4430	20
Petal length (mm)	9.66 ± 0.16	20
Petal width (mm)	6.33 ± 0.16	20
Sepal length (mm)	6.65 ± 0.13	20
Sepal width (mm)	2.39 ± 0.06	20
Corolla diameter (mm)	15.46 ± 0.25	20
Pedicel length (mm)	12.01 ± 0.32	20

### Effects of staminal hair removal on visitation by honeybees

**Figure 2.**
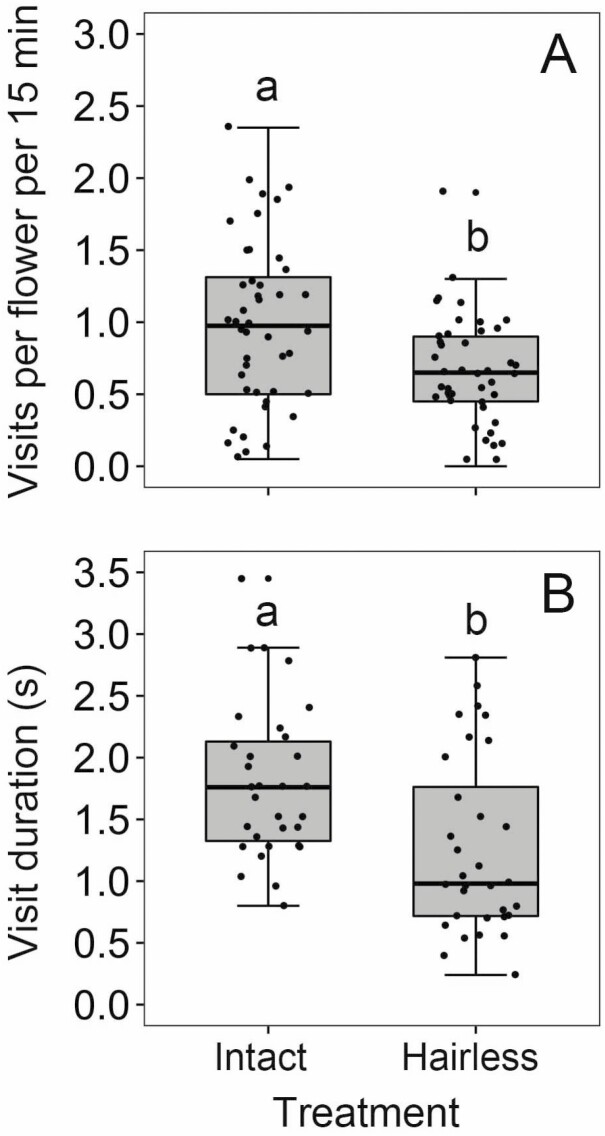
The effects of staminal hair treatments on visitation rates (visits per flower per 15 min) (A) and visit duration (B) of honeybees (*A. cerana*). The box plots indicate the median (mid lines), the interquartile range (boxes) and ×1.5 the interquartile range (whiskers). Different lowercase letters show significant differences at *P* < 0.05.

The GLM analysis showed that the visitation rate of honeybees to control flowers was 0.99 ± 0.09 visits per flower per 15 min (mean ± SE, *N* = 44), which was significantly higher than that to hairless flowers (0.65 ± 0.06 visits per flower per 15 min, *N* = 44, Wald *χ*^2^ = 10.419, *P* = 0.001; Fig. 2A). It suggested that staminal hairs increase the attractiveness of flowers to honeybees in *T. fluminensis*. Moreover, honeybees stayed longer on control flowers (1.79 ± 0.11 s, *N* = 31) than on hairless flowers (1.26 ± 0.13 s, *N* = 32, Wald *χ*^2^ = 9.97, *P* = 0.002; Fig. 2B), suggesting that flowers with staminal hairs were favoured by honeybees.

### Effects of staminal hair removal on pollen deposition and pollen removal by honeybees

**Figure 3.**
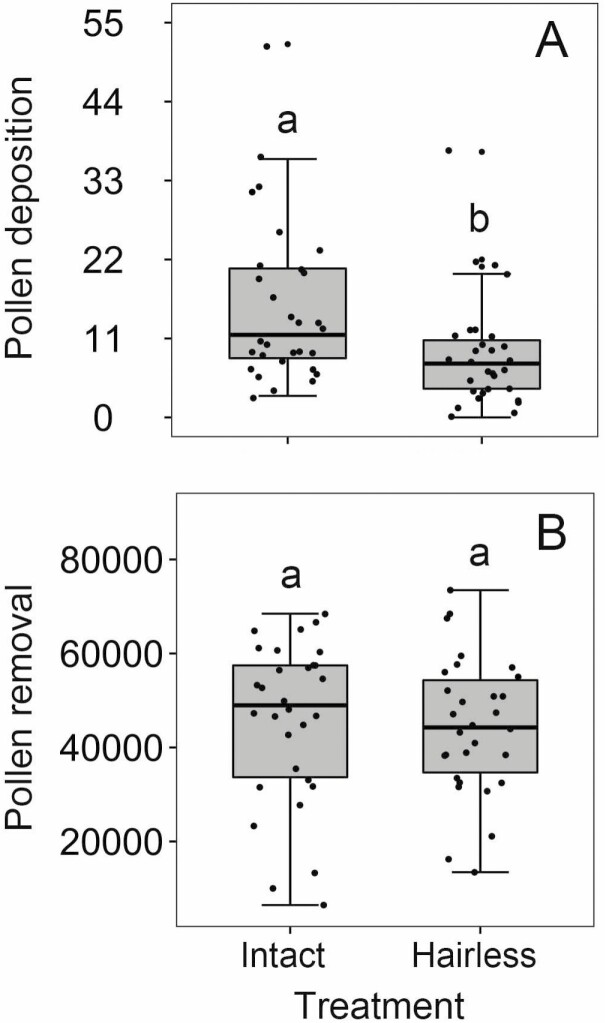
The effects of staminal hair treatments on pollen deposition (A) and pollen removal (B) by honeybees (*A. cerana*). The box plots indicate the median (mid lines), the interquartile range (boxes) and ×1.5 the interquartile range (whiskers). Different lowercase letters show significant differences at *P* < 0.05.

The number of pollen grains deposited on stigmas by a single honeybee visit was significantly more in control flowers (15 ± 2 grains, *N* = 30) than in hairless flowers (9 ± 1 grains, *N* = 30, Wald *χ*^2^ = 8.353, *P* = 0.004; Fig. 3A), indicating that staminal hairs facilitated pollen deposition by honeybees in *T. fluminensis*. However, there was no significant difference in the staminal hair treatments on pollen removal (Wald *χ*^2^ = 0.108, *P* = 0.743; Fig. 3B) and remaining pollen grains (Wald *χ*^2^ = 0.124, *P* = 0.724).

## Discussion

Our investigations on *T. fluminensis* demonstrated that removing the staminal hairs significantly decreased visitation and pollen deposition by honeybees (*A. cerana*), but did not affect pollen removal, only supporting the hypotheses that staminal hairs may attract pollinators and enhance pollination accuracy. Consistent with a previous study on the staminal hairs of *Bulbine abyssinica* in South Africa (Duffy and Johnson 2015), in which the attracting pollinator hypothesis was examined.

Our staminal hair manipulations showed that removing the staminal hairs significantly reduced the visitation rate and visit duration of honeybees (Fig. 2A and B). Several possibilities may explain these results. First, the staminal hairs as advertising structures increase the showiness of the whole floral display in *T. fluminensis*, and thus staminal hairs increase the attractiveness of flowers to honeybees. The staminal hairs of *T. fluminensis* are white and closely surround the yellow anthers, so they attract honeybees by contrasting with the yellow anthers as well. The staminal hairs of *T. fluminensis* may also function as stamen mimicry (Lunau *et al.* 2017; Tagawa 2023), which are similar to the dense stamens of some species (e.g. *Syzygium jambos*) in the family Myrtaceae. Flowering plants present these conspicuous stamens or mimic stamens to attract pollinators (Lunau *et al.* 2007). Second, the flowers with large amounts of staminal hairs provided more surface area and more footholds for honeybees than hairless flowers (Fig. 1E and F), so that flowers with staminal hairs were favoured by honeybees. Third, the last possibility is that honeybees have formed floral constancy to flowers of *T. fluminensis* (Lewis 1993; Wilson and Stine 1996; Gegear and Laverty 2001, 2005). Wilson and Stine (1996) suggested that bees only become sensitized to one morph of a particular floral trait when deciding what flowers to visit. In our study, honeybees may simply become sensitized to staminal hairs of *T. fluminensis*, and thus honeybees show flower constancy to flowers with staminal hairs. In the population of *T. fluminensis*, the staminal hair-removed flowers were comparatively rare and were randomly distributed among the flowers that were intact in appearance. Therefore, when flower-constant honeybees land on the manipulated flowers without staminal hairs, it is most likely to leave early for other familiar flowers which they can skilfully handle.

Furthermore, our results showed that pollen deposition by honeybees in control flowers was significantly more than that in hairless flowers (Fig. 3A). One reason could be that the staminal hairs of *T. fluminensis* affected the posture and behaviour of honeybees on the flowers and increased the accuracy of pollination through the consistent pollen placement and pick-up on honeybees. According to our field observations, when a honeybee collected pollen on the flower with staminal hairs that were 0.72 mm and 0.92 mm shorter than the stamen and the pistil, respectively (Table 1), it lay prone on the staminal hairs. The pollen grains from the anthers were placed on its ventral surface which contacted the stigma during the bee’s next flower visit (Fig. 1E). However, the honeybee had to lie prone on the petals when the staminal hairs were removed (Fig. 1F). In this case, the ventral surface of honeybee rarely contacted the stigma so that fewer pollen grains were deposited. Apart from the reason discussed above, another reason could be that honeybees stayed less time on hairless flowers than on control flowers so that they deposited fewer pollen grains on the stigmas.

The hypothesis that staminal hairs may interfere with pollen collection by bees was not supported by our staminal hair manipulation experiments in *T. fluminensis*, because pollen removal by honeybees on hairless flowers and on control flowers were not significantly different (Fig. 3B). We found that honeybees drew together all of the anthers occasionally before collecting pollen, and they often collected pollen from individual anthers sequentially on both hairless and control flowers. Consequently, the absence of staminal hairs neither affected how bees collect pollen nor increased the efficiency of pollen collection in *T. fluminensis*. In addition, we acknowledge that the statistical power of the analysis is limited given the extreme variability of the pollen count.

Although the previous study has suggested that staminal hairs can enhance plant fecundity (i.e. fruit set and seed set) by increasing the attraction of pollen-seeking insects to flowers in *Bulbine abyssinica* (Duffy and Johnson 2015), they did not consider the role of staminal hairs for pollen transfer. In this study, unfortunately, we did not harvest any fruit of *T. fluminensis*, because the pedicel withered naturally before the fruit matured. Therefore, we did not investigate whether the staminal hairs affect the fruit set and seed set in this species. Future studies could further examine the effects of staminal hairs on reproductive success in other species of *Tradescantia*. In addition, *T. fluminensis*, which originates in South America, is a naturalized species in China (Xu 2022), so the hypotheses regarding the staminal hairs of *T. fluminensis* deserve further test in the original habitats in South America. It may reveal some unique adaptations or specific relationships between *T. fluminensis* and its native pollinators that have evolved in the original habitats.

In conclusion, our experiments provide evidence that staminal hairs play a positive role in attracting pollinators and increasing pollen deposition in *T. fluminensis*. The staminal hairs increase the showiness of the whole floral display and provide more footholds for honeybees. In addition, honeybees lay prone on the staminal hairs to collect pollen, which increased the accuracy of pollination through the consistent pollen placement and pick-up on the ventral surface of honeybees. This study will be helpful for further understanding the adaptive significance of staminal hairs and the role of staminal hairs in maintaining relationships between plants and pollinators.

## Supporting Information

The following additional information is available in the online version of this article –

Appendix S1. Raw data sets involved in this study, including floral traits, visitation rate, visit duration, pollen removal and deposition.

plad067_suppl_Supplementary_Appendixs_S1

## Data Availability

The raw data are available in Appendix S1.
